# Orthotopic heart transplantation for Fontan failure: experience and treatment strategy—a case report

**DOI:** 10.1186/s44215-025-00234-1

**Published:** 2026-01-07

**Authors:** Sakura Horie, Fumiaki Shikata, Yasutaka Hirata, Shigeto Tsuji, Sadayuki Moriyama, Masahiko Ando, Ryo Inuzuka, Hideyuki Kato, Minoru Ono

**Affiliations:** 1 Department of Cardiovascular Surgery, The University of Tokyo Hospital, 7-3-1 Hongo, Tokyo, Bunkyo-Ku 113-8655 Japan; 2 Department of Cardiovascular Surgery, National Center for Child Health and Development, Tokyo, Japan; 3 Department of Pediatrics, The University of Tokyo Hospital, Tokyo, Japan; 4 Department of Cardiovascular Surgery, University of Tsukuba Hospital, Ibaraki, Japan

**Keywords:** Fontan circulation, Fontan failure, Orthotopic heart transplantation, Pediatric heart transplantation, Extracardiac total cavopulmonary connection

## Abstract

**Background:**

Orthotopic heart transplantation after the Fontan operation presents technical surgical challenges due to the connection of systemic veins to pulmonary arteries and well-developed systemic-to-pulmonary collateral arteries. The altered anatomy and hemodynamics necessitate extensive vascular reconstruction. We report a successful orthotopic heart transplantation with three years of ventricular assist device (VAD) support in a child who had undergone the Fontan operation.

**Case presentation:**

A 10-year-old boy had undergone extracardiac total cavopulmonary connection (18 mm expanded polytetrafluoroethylene conduit) at 2 years of age for a large ventricular septal defect, straddling tricuspid valve, and mitral stenosis. Following the Fontan operation, his systemic ventricular function gradually deteriorated. At 7 years of age, a Berlin Heart EXCOR® Pediatric VAD was implanted due to progressive heart failure, and he was listed for heart transplantation. Three years later, a heart transplant was performed.

Cardiopulmonary bypass was established via cervical cannulation before re-sternotomy. The superior vena cava and extracardiac conduit were detached from the pulmonary artery. The pulmonary artery was reconstructed from hilum to hilum with a large bovine pericardial patch. Well-developed systemic-to-pulmonary collaterals caused excessive left atrial return; therefore, the left atrial anastomosis was performed under deep hypothermic circulatory arrest. The systemic veins were reconstructed with bicaval anastomosis, and inferior vena caval continuity was restored by leaving a short segment of the previous conduit. The procedure was completed without complications. Postoperative recovery was uneventful, and the patient was discharged on day 35.

**Conclusions:**

This case illustrates a successful approach to orthotopic heart transplantation in a child with failing Fontan circulation supported by a VAD. Reconstruction of the pulmonary artery using a large pericardial patch and restoration of bicaval continuity were key to overcoming complex anatomical challenges.

## Background

An estimated 70,000 individuals live with Fontan circulation worldwide, of whom 5–10% progress to failing Fontan requiring transplantation [1–3]. Orthotopic heart transplantation after Fontan surgery poses technical challenges—small pulmonary arteries, disconnected systemic veins, dense mediastinal adhesions, and extensive systemic-to-pulmonary collaterals [4]. We report a successful orthotopic heart transplantation with three years of ventricular assist device (VAD) support in a child with who had previously undergone Fontan operation.

## Case report

A boy had a large ventricular septal defect, mitral stenosis, tricuspid-valve straddling, right aortic arch with aberrant left subclavian artery, and a retro-aortic innominate vein. At 2 years of age, Fontan completion was performed with an 18 mm extracardiac ePTFE conduit. Ventricular function gradually declined postoperatively. At age 7, due to worsening heart failure and inotrope dependence, a Berlin Heart EXCOR® was implanted as a bridge to transplantation. He was listed for heart transplantation, which was performed three years later.

## Case presentation

The boy was transferred to our hospital the day before heart transplantation under VAD support. Preoperative laboratory data showed WBC count of 10 × 10^3^/µL and CRP of 1.2 mg/dL, attributed to localized infection at the VAD cannula exit sites. Liver and kidney function were normal. Chest radiography showed cardiomegaly with a cardiothoracic ratio of 68%. Contrast-enhanced CT revealed the inflammatory change around the VAD cannula without other significant findings. The EXCOR VAD cannulation sites were infected, with associated skin defect.

**Fig. 1:**
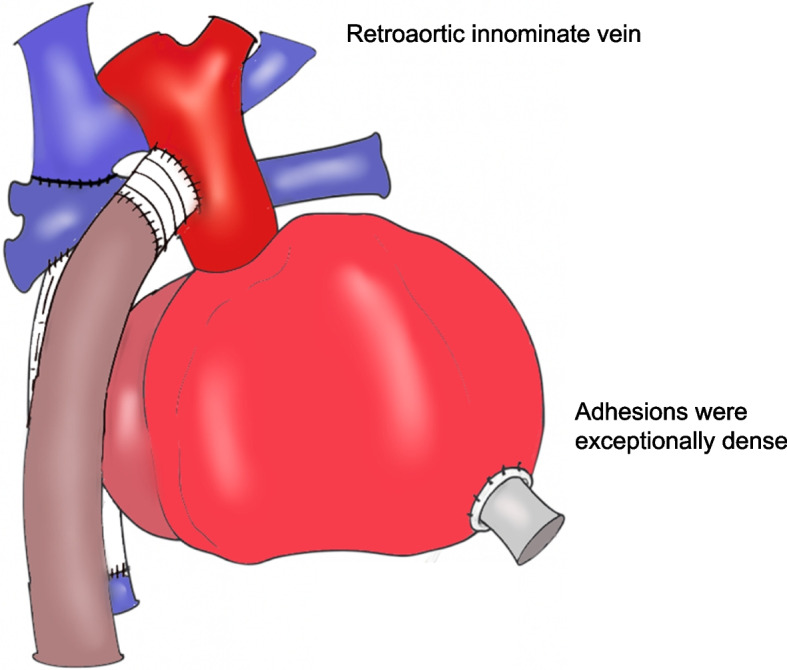
Redo sternotomy revealed dense adhesions, requiring extensive dissection. A retroaortic innominate vein was present. The VAD outflow cannula was in the ascending aorta; inflow was in the right ventricular apex. The Fontan conduit lay dorsal to the outflow cannula

**Fig. 2:**
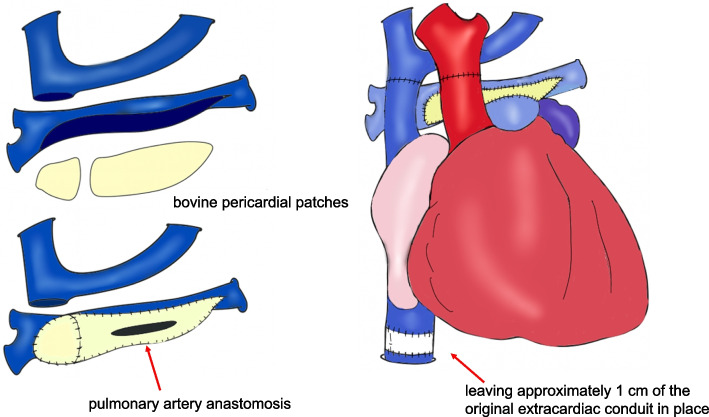
The aorta was cross-clamped and transected. The SVC was detached and an ePTFE graft removed. The pulmonary artery was reconstructed hilum to hilum with two bovine patches. The IVC was short, so ~ 1 cm of conduit was retained. All anastomoses were completed

A transverse incision was made in the right neck. An 8 mm woven graft was anastomosed to the internal carotid artery with 6–0 polypropylene suture to establish arterial inflow for cardiopulmonary bypass. A 20 Fr venous cannula was inserted into the internal jugular vein, and bypass was initiated. The ventilation was stopped before the resternotomy to avoid the lung injury. Adhesions were severe and dissection was time-consuming (Fig. 1). The aorta was cross-clamped and transected. The superior vena cava was detached from the pulmonary artery, and the ePTFE graft beneath the pulmonary artery was removed. The main pulmonary artery was reconstructed from hilum to hilum using two bovine pericardial patches with 6–0 polypropylene suture (Fig. 2).

After trimming the donor heart, the left atrium was anastomosed to the recipient’s cuff using 5–0 polypropylene suture. Due to poor visualization caused by excessive venous return, deep hypothermic circulatory arrest was initiated. Cardiopulmonary bypass resumed after two-thirds of the anastomosis was complete. The inferior vena cava was anastomosed with 5–0 polypropylene, leaving about 1 cm of the original extracardiac conduit, followed by pulmonary artery and aortic anastomoses using running 5–0 polypropylene suture (Fig. 2). After de-airing, the aortic cross-clamp was removed. Inhaled nitric oxide was started at 20 ppm. The superior vena cava was trimmed and anastomosed with running 6–0 polypropylene. One hour after declamping, CPB was discontinued. Hemostasis was confirmed, and infected EXCOR cannula sites were debrided. The total operative time was 10 h and 12 min. The CPB time was 424 min, and the aortic cross-clamp time was 215 min. Deep hypothermic circulatory arrest lasted for 33 min, with a minimum rectal temperature of 20.1 °C. The ischemic time was 249 min, consisting of a cold ischemic time of 147 min and a warm ischemic time of 102 min. Delayed sternal closure was chosen. The chest was closed after wound irrigation the next day. Recovery was uneventful, and the patient was discharged on day 35. He was doing well 10 months after transplantation.

## Discussion

A distinctive feature of this case is the prolonged waiting period for heart transplantation in Japan compared with Western countries. In Western countries, pediatric heart transplantation is typically performed within a few months of listing [5], whereas in Japan, donor scarcity often results in significantly longer waiting times [6]. In the present case, approximately three years elapsed between EXCOR implantation and transplantation. During such an extended waiting period with VAD management, various complications tend to arise, including infection of VAD cannula sites, severe intrathoracic adhesions, loss of central venous access routes associated with multiple surgeries. We anticipated three major challenges in this patient’s orthotopic heart transplantation. First, dense mediastinal adhesions from multiple prior sternotomies, combined with the unique anatomy of systemic veins directly connected to pulmonary arteries, increased the risk of bleeding and lung injury. Second, extensive systemic-to-pulmonary collaterals hindered the clear operative field, complicating exposure and hemostasis. Third, small pulmonary arteries and elevated pulmonary vascular resistance posed a risk of right ventricular failure [7, 8].

Temur et al. reported that cervical cannulation significantly reduces bleeding during re-sternotomy. In this case, grafts were anastomosed to the right common carotid artery and internal jugular vein, and cardiopulmonary bypass was established before re-sternotomy in the lung-down position, minimizing lung injury and hemorrhage despite dense adhesions [9]. Patch augmentation is also effective in patients with hypoplastic pulmonary arteries [10, 11]. Here, hilum-to-hilum enlargement using a bovine pericardial patch allowed tension-free end-to-end donor PA anastomosis, achieving favorable postoperative hemodynamics with a peak velocity < 2.0 m/s, avoiding right heart failure. Konstantinov et al. reported that early deep hypothermia and anastomosis under circulatory arrest reduce bypass time by limiting collateral flow [7]. In this case, circulatory arrest at 20.1 °C for 33 min ensured excellent visualization and stable hemostasis during left atrial anastomosis.

We believe that the meticulous preoperative planning of surgical strategies to address these anticipated issues was a key factor in safely completing heart transplantation after the Fontan procedure, despite complex anatomy and dense adhesions, and in achieving a favorable postoperative course.

## Conclusions

This case demonstrates successful orthotopic heart transplantation in a child with failing Fontan circulation supported by VAD. Reconstruction of the pulmonary artery with a large pericardial patch and restoration of bicaval continuity were essential for managing complex anatomy.

## Data Availability

The data supporting this article are included in the article and available from the corresponding author upon reasonable request.

## Abbreviations

VAD: Ventricular assist device
TCPC: Total cavopulmonary connection
ePTFE: Expanded polytetrafluoroethylene
